# Transforming Drug Development for Neurological Disorders: Proceedings from a Multidisease Area Workshop

**DOI:** 10.1007/s13311-023-01440-x

**Published:** 2023-10-12

**Authors:** Diane Stephenson, Ramona Belfiore-Oshan, Yashmin Karten, Jessi Keavney, D. Kevin Kwok, Terina Martinez, Joe Montminy, Martijn L. T. M. Müller, Klaus Romero, Sudhir Sivakumaran

**Affiliations:** Critical Path Institute, Tucson, AZ USA

**Keywords:** Alzheimer’s disease, Parkinson’s disease, Duchenne muscular dystrophy, Huntington’s disease, Ataxia, Data sharing

## Abstract

Neurological disorders represent some of the most challenging therapeutic areas for successful drug approvals. The escalating global burden of death and disability for such diseases represents a significant worldwide public health challenge, and the rate of failure of new therapies for chronic progressive disorders of the nervous system is higher relative to other non-neurological conditions. However, progress is emerging rapidly in advancing the drug development landscape in both rare and common neurodegenerative diseases. In October 2022, the Critical Path Institute (C-Path) and the US Food and Drug Administration (FDA) organized a Neuroscience Annual Workshop convening representatives from the drug development industry, academia, the patient community, government agencies, and regulatory agencies regarding the future development of tools and therapies for neurological disorders. This workshop focused on five chronic progressive diseases: Alzheimer’s disease, Parkinson’s disease, Huntington’s disease, Duchenne muscular dystrophy, and inherited ataxias. This special conference report reviews the key points discussed during the three-day dynamic workshop, including shared learnings, and recommendations that promise to catalyze future advancement of novel therapies and drug development tools.

## Introduction

There is an urgent need to advance new and innovative therapeutic approaches and drug development tools for neurological disorders. The massive health and economic impacts of neurological diseases have raised the issue to an international health policy level, capturing the attention of the World Health Organization in the most rapidly growing nervous system diseases [1]. Common challenges that are shared across individual neurological diseases include the variable course of disease trajectories, the lack of biomarkers that track the onset and progression of disease, and the need for patient focused endpoints. Such factors contribute to the necessity for long duration and costly clinical trials. Few opportunities exist to share learnings across individual diseases and to encourage collaborations among diverse disease-focused stakeholders around the world. Regulatory agencies across the globe have recommended public–private partnerships as key to accelerating drug development [2–5].

The Critical Path Institute (C-Path) is a unique nonprofit organization with the mission of leading collaborations that accelerate drug development, advancing better treatments for people worldwide. C-Path serves as a neutral third party to lead public–private partnerships (PPPs) for several chronic diseases of high unmet medical need. The range of diseases that impact the nervous system, covered by C-Path PPPs, includes Alzheimer’s disease (AD), Parkinson’s disease (PD), Huntington’s disease (HD), Duchenne muscular dystrophy (DMD), and inherited ataxias. C-Path leads collaborative teams to advance regulatory science needs in many specific disease areas, and neurological disorders are a key focus. To date, C-Path has successfully advanced data-driven approaches to advance drug development tools with regulatory milestones achieved in several disorders of the nervous system. Table 1 lists specific drug development tools that have received FDA and/or EMA regulatory endorsement by C-Path consortia for AD, PD, and DMD. Table 2 illustrates modeling drug development tools under regulatory review for HD, PD, and DMD. The drug development tools listed in Tables 1 and 2 include biomarkers and clinical trial simulation tools all developed by integrating diverse clinical data from around the world. Endorsement of such tools then serves to streamline drug development review for future sponsors that utilize these tools in their programs.

**Table 1:** Regulatory milestones achieved in public private partnerships for nervous system disorders

**Alzheimer’s disease: regulatory successes**				
**2011**	EMA	Qualification	Qualification opinion of low hippocampal volume (atrophy) by MRI for use in clinical trials for regulatory purpose—in pre-dementia stage of Alzheimer’s disease	LINK
**2013**	FDA	Fit for purpose	Disease model of mild to moderate AD	LINK
**2013**	EMA	Qualification	Disease model of mild to moderate AD	LINK
**2015**	FDA	FDA Biomarker Survey	Recommendation of Alzheimer’s biomarkers Neurogranin and Tau PET (*collaboration with Kaj Blennow, Mike Weiner on behalf of CAMD*) in response to: “Biomarker Survey—Identifying Potential Biomarkers for Qualification and Describing Contexts of Use to Address Areas Important to Drug Development”	LINK
**2015**	FDA	Letter of support	Cerebral spinal fluid (CSF) analytes Aβ1-42, t-tau, and p-tau as exploratory prognostic biomarkers for enrichment in AD trials	LINK
**2015**	FDA	Letter of support	Low baseline hippocampal volume as an exploratory prognostic biomarker for enrichment in AD trials	LINK
**2018**	EMA	Letter of support	Model-based clinical trial enrichment tool for clinical trials in amnestic mild cognitive impairment	LINK

**Parkinson’s disease: regulatory successes**				
**2015**	FDA	Letter of support	Exploratory prognostic biomarkers for enrichment in early-stage Parkinson's disease clinical trials; molecular neuroimaging biomarker: dopamine trransporter	LINK
**2016**	EMA	Letter of support	Molecular imaging of the dopamine transporter biomarker as an enrichment biomarker for clinical trials for early Parkinson's disease	LINK
**2018**	EMA	Qualification opinion	Molecular neuroimaging of the dopamine transporter as biomarker to identify patients with early manifest Parkinsonism in Parkinson's disease	LINK
**2019**	FDA	Critical path innovation meeting	Digital drug development tools for early Parkinson’s disease clinical trials	––-
**2019**	EMA	Innovative task force	Digital drug development tools for early Parkinson’s disease clinical trials	––-
**2022**	EMA	Letter of support	Model-based clinical trial simulation platform to optimize design of efficacy evaluation studies in Parkinson’s disease	LINK

**Duchenne muscular dystrophy: regulatory successes**				
**2018**	EMA	Letter of support	Letter of support for glutamate dehydrogenase, a biomarker of hepatocellular liver injury: in collaboration with the Predictive Safety Testing Consortium (PSTC) hepatotoxicity working group at C-Path	LINK
**2022**	EMA	Letter of support	A model-based clinical trial simulation tool to optimize clinical trial design of studies to investigate efficacy of potential therapies for Duchenne muscular dystrophy	LINK

**TBI: regulatory successes**				
**2017**	FDA	Letter of support	Letter of support for the use of MRI to assess cortical contusions and diffuse axonal injury as exploratory prognostic enrichment biomarkers to identify patients that are likely to develop permanent disability during the course of mild traumatic brain injury trials	LINK
**2018**	FDA	Letter of support	The letter of support to the TBI Endpoints Development (TED) initiative and the Transforming Research and Clinical Knowledge in Traumatic Brain Injury (TRACK-TB!) investigators to encourage the further study of blood levels of glial fibrillary acidic protein (GFAP), a possible biomarker of astrocytic injury, and ubiquitin carboxyl-terminal hydrolase Ll (UCH-Ll), a possible biomarker of neuronal injury, as exploratory prognostic enrichment biomarkers to identify patients who are likely to develop persistent disability during the course of mild traumatic brain injury (TBI) clinical trials	LINK
**2019**	FDA	MDDT (device) qualification	Neuroimaging assessment of brain contusions, as assessed by an expert rater from MRI using OsiriX CDE Software Module MDDT, may be used for enrichment of clinical trials for TBI	LINK

Drug development tools that have received regulatory endorsement by FDA and EMA for nervous system disorders. This list comprises examples advanced by C-Path public private partnerships with the exception of TBI which was led by the TED (Traumatic Brain Injury Endpoints Development) consortium with C-Path as a partner organization.

*AD* Alzheimer’s disease, *PD* Parkinson’s disease, *HD* Huntington’s disease, *DMD* Duchenne muscular dystrophy, *TBI* traumatic brain injury, *MDDT* Medical Device Drug Development Tool initiative

**Table 2:** Drug development tool neuroscience initiatives under regulatory review

**C-Path active neuroscience programs under regulatory review**				
**2022**	FDA	Fit for purpose	A Model-Based Clinical Trial Simulation Tool to Optimize Clinical Trial Enrichment and Design of Efficacy Evaluation Studies in Huntington’s Disease	HD-RSC
**2022**	EMA	EMA qualification of novel methodologies	A Model-based Clinical Trial Simulation Tool to Optimize Clinical Trial Design of Studies to Investigate Efficacy of Potential Therapies for Duchenne Muscular Dystrophy	D-RSC
**2022**	FDA	Fit for purpose	A Modeling-based Clinical Trial Simulation Tool focused on Non-invasive Magnetic Resonance Spectroscopy-based Muscle Fat Fraction and Functional Outcome Measures to Optimize Trial Design in Duchenne Muscular Dystrophy	D-RSC
**2023**	FDA	Fit for purpose	A Model-Based Clinical Trial Simulation Tool to Optimize Design of Efficacy Evaluation Studies in Parkinson’s Disease	CPP

Drug development tool projects under review by FDA and EMA (October 2022) in support of drug development tools to support model informed drug development. Disease areas include PD, HD, and DMD

*HD-RSC* Huntington’s Disease Regulatory Science Consortium, *D-RSC* Duchenne Regulatory Science consortium, *CPP* critical path for Parkinson’s consortium

C-Path organized a Neuroscience Annual Workshop convening representatives from academia, industry, regulatory agencies (FDA and EMA), and the patient community to address a range of unmet needs and challenges in drug development (a list of workshop participants is included in the acknowledgments). This diverse set of voices across the ecosystem was critical to generating a holistic output of perspectives including the patient voice to be shared with the wider community and distilled into recommendations for the future.

## Patient-Focused Drug Development: the Patient Voice Drives Change

The 21st Century Cures Act statute (85 FR 25642 [34]) specified that the FDA develops guidance documents over a period of five years regarding the collection of patient experience data and the recommendations for the proper use of such data and related information in the process of drug development. This initiative is referred to as patient-focused drug development (PFDD) and is grounded in four FDA guidance documents (https://www.ema.europa.eu/en/events/multi-stakeholder-workshop-patient-experience-data-medicines-development-regulatory-decision-making). Individuals living with a disease are true experts with lived experience and are uniquely positioned to inform the therapeutic context for evaluation of safety and efficacy of new drugs under development. A systematic approach led by regulatory agencies has been transformative to ensure that patients’ experiences, perspectives, needs, and priorities are captured and meaningfully incorporated into the drug development and evaluation processes [6, 7]. Perspectives from five annual meeting in-person participants who represented the patient voice were shared throughout the three days of the workshop. The lived experience of individuals affected by AD (patient perspective Box 2) and PD (patient perspectives Box 1 and Box 3) provided a unique sense of urgency and inspiring viewpoints for all to learn from.

## Clinical Outcome Measures as Clinical Trial Endpoints

Traditional endpoints to study progression of neurological disorders primarily rely on measures assessed by clinicians evaluating signs and symptoms based on impact and individuals’ inability to perform functional tasks in their daily lives. Yet there are unique differences between endpoints used in clinical settings as compared to what is required for evaluation of safety and efficacy of new drugs. The implementation of PFDD has catalyzed the recognition of improved measures that are reflective of the patient and caregiver voice. This has led to the emergence of new or refined clinical outcome assessments (COAs) for use in clinical trials. Patient organizations and patient representatives play an integral role in developing COAs. It is recognized that neurological diseases represent a continuum rather than a defined list of discrete milestones and that a time-to-event endpoint might not be adequate for chronic progressive disorders where the pathophysiology of disease occurs over decades. Multiple regulatory pathways are in place to advance COAs for use in clinical trials. It is important to distinguish between evaluation of signs and symptoms in clinical care vs. a well-defined COA needed for evaluation of clinical trials. FDA’s PFDD guidance has been transformative in defining the requirements for fit-for-purpose COAs of clinical trials. The EMA recently held a “Multi-stakeholder workshop: Patient experience data in medicines development and regulatory decision-making” (European Medicines Agency, 2022; https://www.ema.europa.eu/en/events/multi-stakeholder-workshop-patient-experience-data-medicines-development-regulatory-decision-making) with the goal of highlighting the importance of including the patient voice in regulatory review of medical products in the European Union. Both the FDA and the EMA are open to novel approaches and endpoints, particularly for diseases where there is no precedent.

## Understanding Disease Progression for Optimizing Endpoints

Global health authorities have identified natural history studies and data from registries as suitable supporting data for drug approvals, particularly in orphan diseases [8, 9]. Strategies include the use of natural history to generate historical control data for a range of applications such as in silico simulations, use of external controls, nontraditional study designs, and identifying inclusion/exclusion criteria and appropriate endpoints from untraditional data sources. These examples have been captured in recent regulatory guidance documents publicly posted on behalf of both FDA and EMA.

**Fig. 1:**
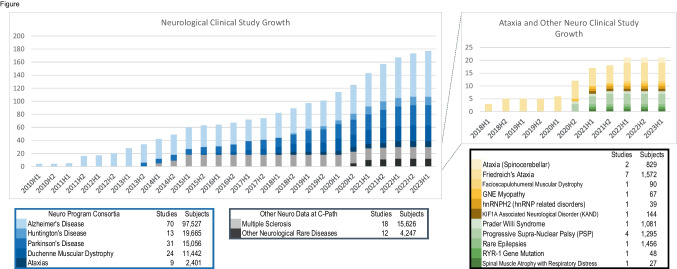
Graphic histogram of the data acquired and integrated into unified databases at C-Path across distinct diseases that impact the nervous system. Patient-level item level data is fully anonymized and integrated using CDISC therapeutic area standards. The number of participants denotes the status as of January 2023

C-Path is a leader in data aggregation, standardization, and generation of hypotheses and solutions based on patient-level and item level data [10] across diverse sources of clinical data. Most of the neurology data sets in the C-Path repository are from industry clinical trials, constituting high-quality controlled data of the highest standards and rigor and are well curated making them suitable for modeling and analyses that can accelerate and increase efficiency in drug development (Fig. 1).

## Digital Health Technologies as Drug Development Tools

The integration of digital health technologies (DHTs) into drug development is advancing at a rapid pace [11]. A broad spectrum of applications for DHTs has emerged including early diagnosis, longitudinal characterization, and monitoring of disease progression. The ability to derive continuous measures of daily life function in real-world settings holds significant promise for decentralized trials in neurological diseases. Confidence in the reliability and reproducibility of the measures derived from DHTs is essential to assure that the platform is fit-for-purpose and to effectively advance the successful integration of DHTs in drug development. Cutting edge advances in technology platforms, algorithm development, and robust analytic platforms pose both advantages and challenges given the rapid pace of innovation. Independent validation of study results is still lacking in the majority of case examples. Several regulatory frameworks have been proposed, and it is recommended that early and close communication with regulatory health authorities is followed to ensure that the validation plan will address evidentiary requirements, which allow for integration of DHTs in drug development. The use of such tools as exploratory endpoints in clinical trials and sharing of the generated data promises to accelerate the rate of progress in the field. This requires multidisciplinary stakeholders across a diverse array of disciplines to collaborate within a pre-competitive framework to achieve success. Integrating patient perspectives is especially critical at all stages of DHT development, study design, and execution. The Critical Path for Parkinson’s (CPP) digital drug development tool (3DT) initiative was highlighted as a case example that is unique in sharing of data, costs, and knowledge under the iterative advisement of regulators. A dedicated team comprised of industry, academic experts, patient research organizations, clinicians, regulators, and people living with PD have agreed to collaborate on prospective data collection and share data to advance the regulatory endorsement of DHTs for use in PD clinical trials [12, 13].

## Biomarkers

Throughout the three days of the annual meeting, regulators emphasized the importance of understanding the biology of a given disease to better understand its various stages and advised implementation of tools to measure its progression. A central message was that a disease should not be exclusively defined by its clinical manifestations but also should be defined by the biology. The syndromic landscape of neurodegenerative diseases is shifting to one that includes more precisely grouped subtypes with diverse molecular pathologies. AD represents a flagship example that has shifted from postmortem confirmation of diagnosis as gold standard to premortem classification that incorporates molecular neuropathological hallmarks of disease such as in vivo measurement of β-amyloid, hyperphosphorylated tau, and TDP-43. Biomarker classification has catalyzed biological staging of disease and incentivized early intervention in AD. Similarly, a new HD Integrated Staging System (HD-ISS) based on biomarkers and genetics was developed by C-Path’s HD Regulatory Science Consortium (HD-RSC) [14]. To consider biomarkers as primary data supportive of drug approval is a relatively new concept in neuroscience. The work at C-Path provides tremendous opportunities to advance overall science towards using biomarkers to capture the underlying disease biology in patients and to implement these evolving insights into drug development in dynamic and iterative ways.

Recent regulatory approvals for disorders that impact the nervous system represent true paradigm shifts in many ways from historical approaches. The acceptance of a greater degree of uncertainty with robust scientific protocols and rigorous assessment of the data is a prerequisite. One example is accelerated approval paths which provide a regulatory pathway to make therapies available to patients with serious life-threatening diseases for which there are no therapies earlier than the more traditional regulatory pathways might allow. In neuroscience, there are, however, significant barriers to applying accelerated approval in regulatory decision-making. A major challenge is the need for biomarkers that reliably reflect the disease biology or intermediate endpoints that reasonably predict clinical benefit. The case of amyloid as a likely surrogate of efficacy for drugs to slow disease progression in early stages of AD was highlighted as an example of the ability to rely on biomarkers to make regulatory decisions [15]. This decision was grounded in an understanding of the disease stages as defined by biomarkers [16]. Additional examples include the role of neuroimaging biomarkers in defining the longitudinal progression of HD [17, 18] and neurofilament light chain (NfL) as a reasonably likely surrogate biomarker in ALS [19].

### Fluid Biomarkers

Recent advances in the measurement of biomarker analytes in cerebrospinal fluid and blood are having significant impact on drug development and leading to a better-informed decision-making. The ability to measure pathologic proteins such as mutant Huntingtin, amyloid, tau, and alpha-synuclein with ultrasensitive assays in biologic fluids is advancing rapidly. Proteinopathies are now being pursued for therapeutic intervention across a range of disorders previously assumed to be distinct disease states due to diverse clinical manifestations (e.g., frontotemporal dementia and amyotrophic lateral sclerosis). Multistakeholder attention to assay standardization, harmonization, prospective integration, and rigorous longitudinal assessment of these promising biomarkers in natural history studies is critical.

The FDA has issued multiple letters of support for biofluid biomarkers as a regulatory path to identifying promising tools for drug development. Examples include the blood biomarkers GFAP and UCHL1 for traumatic brain Injury(https://www.fda.gov/media/112687/download) [20] and NfL in progressive multiple sclerosis (MS) (https://www.fda.gov/media/149608/download) [21]. The letter of support mechanism exists with both FDA (https://www.fda.gov/drugs/biomarker-qualification-program/letter-support-los-initiative) and EMA (https://www.ema.europa.eu/en/human-regulatory/research-development/scientific-advice-protocol-assistance/novel-methodologies-biomarkers/opinions-letters-support-qualification-novel-methodologies-medicine-development) and has led to an increase in use of such biomarkers in clinical trials and facilitates more data collection and data sharing. The letters of support serve as catalysts to further drug development and enable alignment for a more unified consensus on which promising biomarkers should be measured and evaluated in ongoing and future trials. A list of biomarkers that were highlighted during the workshop is illustrated in Table 3.

**Table 3:** Examples of biomarkers and data sharing reviewed at the C-Path neuroscience meeting

**Specific case examples highlighted at C-Path neuroscience conference**		
	*Biomarkers*	*Data sharing examples*
AD	ptau 271, TauPET, amyloid PET, GFAP	AMYPAD, ADNI, GAAIN
		DIAN -TU
		MMission AD
		GENERATION
	Centiloid, CenTauR harmonization	
PD	Dopamine transporter (DAT) SPECT	
PPMI	alpha-synuclein CSF seeding assay	
HD	Mutant Huntington, NfL, vMRI	
DMD	Imaging	
	Digital health technologies	

Table of biomarkers and data sources that were shared by participants at the Neuroscience Annual meeting as examples of successful data sharing

*ptau271* phosphorylated tau at amino acid 271, *Tau PET* Tau positron emission tomography neuroimaging, *AMYPAD* amyloid imaging to prevent Alzheimer’s disease, *ADNI* Alzheimer’s disease neuroimaging Initiative, *GAAIN* Global Alzheimer’s Association Interactive Network, *PPMI* Parkinson’s Progressive Marker Initiative, *DIAN-TU* dominantly inherited Alzheimer’s network, *MissionAD* investigational oral BACE (beta amyloid cleaving enzyme inhibitor in patients with early Alzheimer’s disease (AD), *GENERATION* CAD106 and CNP520 to prevent or delay symptoms of Alzheimer’s disease (Novartis), *SPECT* single photon emission computed tomography, *vMRI* volumetric magnetic resonance imaging

### Imaging Biomarkers

The ability to identify and quantify in vivo the hallmark pathological markers amyloid and tau has transformed drug development for AD [22, 23]. The potential for imaging biomarker modalities such as positron emission tomography (PET) as drug development tools is unique, as it allows for defining and quantifying brain region-specific changes that may correlate with functional outcomes. While a powerful tool, the neuroanatomic spatial specificity of neuroimaging biomarkers, cannot be achieved by biofluid measurement of specific analytes in blood or cerebral spinal fluid (CSF). Neuroimaging biomarkers have the potential to predict earlier symptom onset for individual patients and to assess longitudinal progression with region-specific neuroanatomic precision (e.g., [24]). Imaging of biomarker modalities outside the brain may be informative, in particular early in disease, when autonomic dysfunction may occur or, for example, where the enteric nervous system has been hypothesized to play a role in the etiology of neurological disease, as in PD. Quantitative magnetic resonance imaging (MRI) and spectroscopy (MRS) play important roles as well in imaging of neurological disorders (e.g. [18, 25–27]). In DMD, peripheral imaging using MR has been informative to measure muscle damage, inflammation, and fat fraction infiltration [28, 29].

## Advanced Modeling and Analytics

Development of models that are refined based on emerging data is key, and the FDA recommends defining best practices for prospective modeling technologies to integrate contemporary data as new measurement platforms evolve (e.g., [9]). Disease progression models are key to designing and optimizing clinical trials. The last two decades have catalyzed a rapid growth and expansion of model informed drug development (MIDD). Models are evolving for optimizing clinical trial designs in addition to their role in characterizing safety and supporting evaluation of effectiveness of novel therapies. Methodologies include empirical, semi-mechanistic, and mechanistic modeling.

It is important to recognize that one study, whether it be a clinical trial or a natural history study, is not likely to be sufficient to support the true predictive accuracy of a disease progression model for future trials. From a regulatory perspective, merging multiple data sources is key when trying to increase the analytical power of each dataset and to improve descriptions of disease trajectories. Additional methodologies such as quantitative systems pharmacology and item response theory (IRT) modeling can facilitate increased precision in linking novel biomarkers and genes to clinically meaningful domains, particularly in heterogeneous disease conditions.

Both FDA and EMA have elucidated formal regulatory paths for drug developers, sponsors, and regulatory scientists to engage in specific MIDD-based quantitative opportunities in drug development in a real-time manner. The FDA fit-for-purpose (FFP) path was formed in 2013 with the regulatory endorsement of the first clinical trial simulation tool in Alzheimer’s disease as a precedent for other disease areas to follow [30]. The EMA has adopted the qualification of novel methodologies path for quantitative disease progression models. C-Path neurological disease-focused consortia have received two letters of support for the use of clinical trial simulation platforms to optimize the design of clinical trials in PD and DMD (Table 1a).

## Innovative Clinical Trial Designs

Regulatory agencies have served as catalysts to drive innovative clinical trial designs, particularly following the global COVID-19 pandemic. The first adaptive trial was initiated for breast cancer, and the ISPY2 trial is viewed as transformative in enabling collaboration across traditional boundaries between regulators, researchers, and industry partners [31]. Multi-arm adaptive platform trials represent a novel way to evaluate multiple targets with a shared placebo group to enable iterative investigation of novel mechanisms in parallel. Such an approach is particularly attractive for rare diseases. A number of examples are now emerging across neurological disorders including ALS (HEALEY ALS), AD (DIAN-TU), DMD master protocol [32], and PD (path to prevention P2P platform trial in prodromal PD) [33]. In all examples, multistakeholder collaborations are in place to advance the platform trial. Shared learnings across these disease areas are key to improving efficiencies based on key lessons learned from these innovative trials.

Nonprofit research organizations are key in enabling much needed resources as well as providing patient perspectives and facilitating recruitment and other essential clinical resources. Regulators observe that platform trials represent a unique learning opportunity and recommend that such studies are best suited for an initial assessment that is as informative as possible, perhaps testing out new strategies and techniques followed up by confirmation elsewhere.

## Paving the Path for the Future: Outlook and Critical Success Factors

A rich pipeline of disease modifying therapies is advancing rapidly across a broad range of nervous system disorders. The rapid pace of scientific advances in the neurosciences is transforming traditional drug development approaches to enable new pioneering precision medicine strategies grounded in genetics, biomarkers, and innovative technologies. The regulatory landscape globally is innovating by expanding focus on patient focused drug development and clearly pointing the way to hope for drug approvals for disorders that historically had no effective treatments.

Recommendations for the future that emerged from this unique workshop centered around the importance of fostering collaborations among experts across distinct diseases. Forums such as this multistakeholder workshop serve as enablers for achieving consensus on cross-cutting data-driven approaches to solving problems that drug developers face. Progress in drug development tools including biomarkers, innovative clinical trial design, disease progression models, and clinically meaningful endpoints will be hastened by adopting efficient data sharing and by expanding the precompetitive space. It behooves all stakeholders to support data sharing as an ethical imperative as study participants are putting themselves at risk to contribute to science. Attention to adopting unified data standards and inclusion of exploratory tools in early clinical development will streamline the path for drug approvals. Drug development speed is crucial for patients, physicians, and drug development stakeholders alike. Regulators serve as catalysts for driving change for the future with urgency in meeting the needs of all those impacted by such devastating diseases.

## Voice of the Patient Perspectives

Box 1 D. Kevin Kwok PharmD (Parkinson’s patient) In 2015, as part of the 21st Century Cures Act, I had the privilege of providing testimony to the FDA on the patient experience of living with Parkinson’s Disease. Back then, I was honored to be an early part of the development of these FDA patient engagement guidelines, but I must admit I was uncertain of where this testimonial would go.

In 2022, I was again invited to be a patient participant at the Critical Path/FDA organized Neuroscience Annual Workshop. I opened with my comments that in the seven years that had passed, I was a different Parkinson’s patient with very different needs and issues. It was immediately clear to me that this continuum of neurologic disease was both appreciated and respected by Dr. Billy Dunn and his regulatory colleagues at the FDA and EMA. The responding remark from Dr. Dunn was the need to re-classify the staging of neurologic disease by biology, and not symptoms: This was music to our ears as people living with disease hoping for disease modifying interventions.

I was struck by the partnership the FDA/EMA had with my fellow patients that attended this meeting. In a biopharma industry that has historically focused on Key Opinion Leaders (KOLs), we were valued as POLs (Patient Opinion Leaders). We were invited to have a seat at the table.

For the next 3 days, as POLs, we were active participants in a series of workshops that included designing meaningful clinical outcome measures, understanding disease progression, incorporating DHTs into research, receiving updates on fluid and imaging biomarkers, designing innovative clinical trials and the overarching theme on the importance of data sharing. The patient input we offered was not only encouraged but it was valued, with our remarks often becoming a point of focus for the workshop discussion.

I am by nature a healthy cynic over the acceptance of patient involvement in PFDD. I have experienced firsthand where PFDD is a trendy buzzword and merely only incorporated when convenient or as a PR box-checking exercise for late-stage endorsement. These Critical Path workshops with regulatory agencies and industry sponsors demonstrated that there was a real change evolving in front of us. The participants of this Workshop understood the urgency that those of us with neurodegenerative diseases have.

I traveled home after this October 2022 meeting, thoroughly impressed at the evolution of patient engagement since the seven years of my first FDA presentation. We are at an inflection in Neurosciences R&D and patients can play a direct and important role as part of this team.

Box 2 Joe Montminy (AD patient advocate)  I was impressed to see how the FDA and C-Path are working with industry and drug development experts to examine the gaps and unmet needs in patient-focused drug development (PFDD) in neurology and to incorporate the voice of persons living with a disease into clinical trial designs and the drug development process to generate—and integrate—actionable solutions that benefit everyone.

I felt they were truly listening to what we had to say. We were able to share our perspectives on what PFDD meant to us, our personal experience with PFDD, and what we saw as the benefits of engaging patients in the drug development process. We also discussed the benefits new treatments would have for us and our families. Throughout the meeting, attendees would solicit our input into various issues to help them better understand how these issues impact us. Going into the meeting, I assumed that they just wanted to include the voice of someone living with younger-onset Alzheimer’s disease. I came out of the meeting energized – feeling that they actually wanted to partner with those of us living with a disease to help them improve the clinical trial and drug development process.

To keep this momentum going, drug developers need to engage persons living with a disease throughout the entire drug development and clinical trial process. This will enable them to make the necessary adjustments along the way which can save them time and money. Persons living with a disease can help researchers better understand exactly what benefits we are looking for, which benefits provide the most value to persons living with a disease, and what risks we are willing to take.

We also need to build better trust and relationships between patients and the medical community in underrepresented diverse populations in order to get better representation from these communities. This could encourage more diverse participation in the clinical trials – representative of those it will benefit.

I also feel that increased utilization of technology (online surveys, Zoom, Apps, wearable devices like Apple Watches and Fit Bits, etc.) will increase participation in clinical trials since there are current challenges with participation due to transportation constraints.

Box 3 Jessi Keavney (Parkinson’s advocate) I do not have a formal diagnosis, yet my perspective on treating and preventing neurodegenerative diseases is worth learning from. I have a strong family history of Alzheimer’s and Parkinson’s on both sides of my family, I’m genetically at-risk for Parkinson’s as an LRRK2 G2019S carrier, I’m a former and current care partner to two people with Parkinson’s, and personally most motivating to me is that I’m a mother to three sons who are also at increased risk for NDD. Participating in research is one way that my family honors our past and copes with the uncertainty of the future. Throughout the last nine years, I have participated in over thirty biomarker and observational research studies.

I always appreciate the opportunity to share my research experiences with the hope of improving the long and expensive drug development system. Data sharing, cross-functional collaboration, and returning information to participants in studies are topics that I am particularly passionate about. People can achieve goals quicker when working transparently, and I want to squeeze every ounce of utility from the mounds of data my research contributions have generated over the years. Time and resources cannot be wasted. Consortiums such as C-Path are uniquely positioned to facilitate these objectives, and I was especially enthusiastic to attend and speak on the important topic of data sharing at the Neuroscience annual meeting.

It is not enough to just have a seat at the table. The voices of patients, care partners, and individuals at risk for neurodegenerative diseases should have a fair amount of weight in decisions that impact design, implementation, and outcomes of studies. We can tell when our involvement is tokenistic and empty, but that was not the case at this meeting. It is clear to me that the message from regulatory agencies is that input from patients and their families is mandatory. Even so, our desire is that stakeholders realize that following our suggestions and valuing our feedback from the beginning ultimately benefits everyone, even if it is not a requirement. I came away from the meeting with the overall impression that regulators, industry representatives, academics, and funders are not only listening but also eager to apply direct wisdom from patient advocates.

## Data Availability

Critical Path Institute is committed to data sharing and makes data available for our integrated databases to external qualified researchers.
