# Correction: Transforming Drug Development for Neurological Disorders: Proceedings from a Multidisease Area Workshop

**DOI:** 10.1007/s13311-023-01453-6

**Published:** 2023-11-02

**Authors:** Diane Stephenson, Ramona Belfiore-Oshan, Yashmin Karten, Jessi Keavney, D. Kevin Kwok, Terina Martinez, Joe Montminy, Martijn L. T. M. Müller, Klaus Romero, Sudhir Sivakumaran

**Affiliations:** Critical Path Institute, Tucson, AZ USA

**Correction to: Journal of Neurotherapeutics** **https://doi.org/10.1007/s13311-023-01440-x**

The article“Transforming Drug Development for Neurological Disorders: Proceedings from a Multidisease Area Workshop”, written by Diane Stephenson, Ramona Belfiore-Oshan, Yashmin Karten, Jessi Keavney, D. Kevin Kwok, Terina Martinez, Joe Montminy, Martijn L. T. M. Müller, Klaus Romero, and Sudhir Sivakumaran, was originally published electronically on the publisher’s internet portal on 12 October 2023 without open access. With the author(s)’ decision to opt for Open Choice, the copyright of the article changed on 27 October 2023 to © The Author(s) 2023 and the article is forthwith distributed under a Creative Commons Attribution 4.0 International License, which permits use, sharing, adaptation, distribution and reproduction in any medium or format, as long as you give appropriate credit to the original author(s) and the source, provide a link to the Creative Commons license, and indicate if changes were made. The images or other third party material in this article are included in the article's Creative Commons license, unless indicated otherwise in a credit line to the material. If material is not included in the article's Creative Commons license and your intended use is not permitted by statutory regulation or exceeds the permitted use, you will need to obtain permission directly from the copyright holder. To view a copy of this license, visit http://creativecommons.org/licenses/by/4.0/.

